# Hippocampal, Microglial, Morphological, and Amyloid Profiles Following Thiamine Pyrophosphate Treatment in 3xTg-AD Mice

**DOI:** 10.3390/ijms27115022

**Published:** 2026-06-02

**Authors:** Nelly Jovana Pastén-Castrejón, Humberto Martínez-Orozco, Gloria Yareli Gutiérrez-Silerio, Hebert Luis Hernández-Montiel, Juan Pablo Maya-Arteaga, Israel Poblano-Paez, Pablo García-Solís, Sofía Yolanda Díaz-Miranda

**Affiliations:** 1 Laboratorio de Endocrinología y Nutrición, Facultad de Medicina, Centro de Investigación Biomédica Avanzada de la Universidad Autónoma de Querétaro, Santiago de Querétaro 76140, Mexico; jovana.pasten.08@gmail.com (N.J.P.-C.); gloria.gutierrez@uaq.mx (G.Y.G.-S.); 2 Departamento de Neurobiología del Desarrollo y Neurofisiología, Instituto de Neurobiología-UNAM Campus Juriquilla, Boulevard Juriquilla 3001, Querétaro 76230, Mexico; h_martinez@live.com.mx (H.M.-O.); juanpablo.maya@hotmail.com (J.P.M.-A.); israelpoblano9015@outlook.com (I.P.-P.); 3 Laboratorio de Neurobiología y Bioingeniería Celular, Facultad de Ciencias Naturales de la Universidad Autónoma de Querétaro, Querétaro 76230, Mexico; hebert@uaq.mx

**Keywords:** vitamin B1, Alzheimer’s genetics, microglial morphology, neuroinflammation, amyloid-β clearance, metabolic flexibility, gene expression, osmotic pump, Alzheimer’s disease treatment, mitochondrial dysfunction

## Abstract

Alzheimer’s disease (AD) is characterized by the accumulation of amyloid-β (Aβ) and chronic neuroinflammation, with microglia playing a central role in its pathogenesis. Alterations in microglial metabolism have been proposed to contribute to AD-related inflammatory responses and reduced Aβ clearance, suggesting that thiamine-dependent pathways may be relevant in this context. Thiamine pyrophosphate (TPP), the active form of vitamin B1, is essential for glucose metabolism and mitochondrial function; however, its association with microglial changes in AD remains unclear. In this study, 9-month-old female triple-transgenic AD (3xTg-AD) mice and non-transgenic controls (NoTg) received TPP (2.0 mg/mL) or saline as a vehicle for six weeks via osmotic pumps. Nesting, a hippocampus-dependent behavioral test, as well analyses of Aβ burden, microglial morphology, and the expression of genes related to metabolic and immune pathways were evaluated. Differences in nesting behavior between experimental groups were observed, but TPP treatment was not associated with an evident change in 3xTg-AD mice. In the subiculum and CA1 regions of the hippocampus of female 3xTg-AD mice exposed to TPP, a lower Aβ burden was observed, and morphological variations in microglia were detected in both groups (3xTg-AD and NoTg). Additionally, in the brain of the TPP-treated group, some changes in mRNA gene expression were recorded. Together, these findings describe hippocampal microglial and amyloid profiles following TPP treatment in 3xTg-AD mice and provide a basis for further investigation of thiamine-dependent pathways in AD-related neuroinflammatory contexts.

## 1. Introduction

Alzheimer’s disease (AD) is a neurodegenerative, progressive, and irreversible disorder that affects millions of people worldwide. It is also the main type of dementia in older populations [1]. AD is characterized by amyloid-β (Aβ) accumulation, hyperphosphorylation of tau protein, and chronic neuroinflammation. Together, these alterations are associated with progressive synaptic and cognitive dysfunction [2,3,4]. Moreover, microglia play an important role in AD progression by regulating inflammatory responses and modulating pathways related to neuronal maintenance, metabolism, and homeostasis [5,6].

In AD, microglia contribute to both Aβ plaque formation and clearance throughout disease progression [7,8]. They recognize and phagocytose aggregated Aβ species through several pathways, including those involving the Triggering Receptor Expressed on Myeloid Cells-2 (TREM2) [8,9]. TREM2 is highly involved in the transition of microglia toward a disease-associated microglia (DAM) state [10,11,12]. This transition is accompanied by morphological and transcriptional changes, including reduced process complexity, enlarged soma morphology, and the emergence of reactive microglial states associated with decreased expression of homeostatic markers and increased expression of disease-associated markers, including *Trem2* and *Lgals3,* which have been associated with Aβ plaque formation in AD [13,14].

Aβ accumulation is accompanied by inflammatory and metabolic changes in microglia, including altered TREM2-related signaling and increased expression of mediators such as TNF, IL-1β, IL-6, and iNOS [5,14,15,16]. In addition, the inflammatory state acquired by microglia in AD has been linked to alterations in cellular energy metabolism, including pathways related to glycolysis, mitochondrial function, and TREM2-associated signaling [10,14,17]. These metabolic changes involve enzymes such as PDK1 (*pyruvate dehydrogenase kinase 1*), which regulates *pyruvate dehydrogenase* activity and has been implicated in the balance between glycolysis and oxidative phosphorylation [18,19].

Alterations in microglial energy metabolism have also been proposed in AD. Previous studies suggest that reduced metabolic flexibility may be associated with impaired Aβ clearance and enhanced inflammatory responses, making thiamine-dependent pathways of interest in this context [14,17]. Thiamine pyrophosphate (TPP), the active form of vitamin B1, is an essential cofactor for enzymes involved in glucose metabolism and mitochondrial function [20], including *pyruvate dehydrogenase* and *α-ketoglutarate dehydrogenase* [21,22,23,24,25]. Altered metabolism has also been described in AD, including reduced activity of Thiamine-dependent enzymes and lower expression of *thiamine pyrophosphokinase 1* (TPK1) in affected brain regions [25,26,27].

Experimental studies have shown that TPP participates in pathways related to mitochondrial respiration, oxidative stress, and glucose metabolism [25,28,29]. Because alterations in these processes have been described in AD, TPP has been proposed as a molecule of interest in neurodegenerative conditions [20,30,31]. However, although previous studies have examined thiamine supplementation in AD models, the association of TPP exposure with microglial morphology, inflammatory and metabolic gene expression, and AB burden remains incompletely understood, particularly in early-affected hippocampal regions such as the subiculum and CA1 [31,32].

Therefore, the present study evaluated the association between chronic TPP exposure and hippocampal-dependent nesting behavior, Aβ burden, microglial morphology, and the expression of selected genes involved in inflammatory and metabolic pathways in the hippocampus of 9-month-old female 3xTg-AD mice.

## 2. Results

### 2.1. Hippocampal-Dependent Nesting Behavior Following TPP Exposure in 3xTg-AD Mice

**Figure 1:**
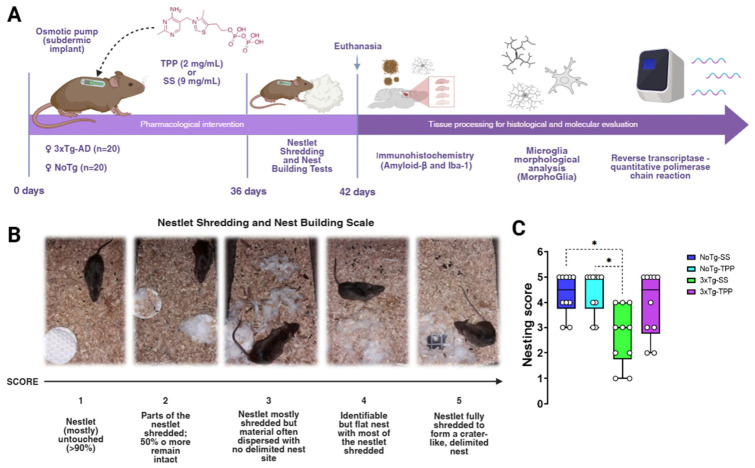
Hippocampal-dependent nesting behavior following TPP exposure in female 3xTg-AD mice. (**A**) Experimental timeline and schematic representation of the TPP intervention protocol. Nine-month-old female NoTg and 3xTg-AD mice received saline solution (SS, 9 mg/mL) or thiamine pyrophosphate (TPP, 2 mg/mL) via osmotic pumps for 42 days. (**B**) The nestlet shredding and nest-building scale, as described by Deacon and Dorninger [33,34], were used to assess nesting behavior. (**C**) Box and whisker plot showing nesting scores of NoTg-SS, NoTg-TPP, 3xTg-SS, and 3xTg-TPP mice. Data are presented as median and interquartile range. Asterisks indicate significant pairwise differences identified by Dunn’s post hoc test following Kruskal–Wallis analysis (* *p* < 0.05). *n* = 10 animals/group. Abbreviations: NoTg, non-transgenic; SS, saline solution; TPP, thiamine pyrophosphate.

Nesting behavior was assessed as a hippocampal-dependent task. After six weeks, 3xTg-SS mice showed lower nest scores than both NoTg groups (*p* = 0.031 vs. NoTg-SS; *p* = 0.014 vs. NoTg-TPP), whereas nest scores in 3xTg-TPP mice were similar to those observed in NoTg-SS and NoTg-TPP animals (*p* > 0.99 for both comparisons). No difference was detected between 3xTg-TPP and 3xTg-SS mice (*p* = 0.179). Overall group differences were supported by the Kruskal–Wallis test (H (3) = 11.49, *p* = 0.0093) followed by Dunn’s post hoc test (Figure 1A–C).

### 2.2. Reduced Hippocampal Aβ Burden Associated with TPP Exposure in 3xTg-AD Mice

**Figure 2:**
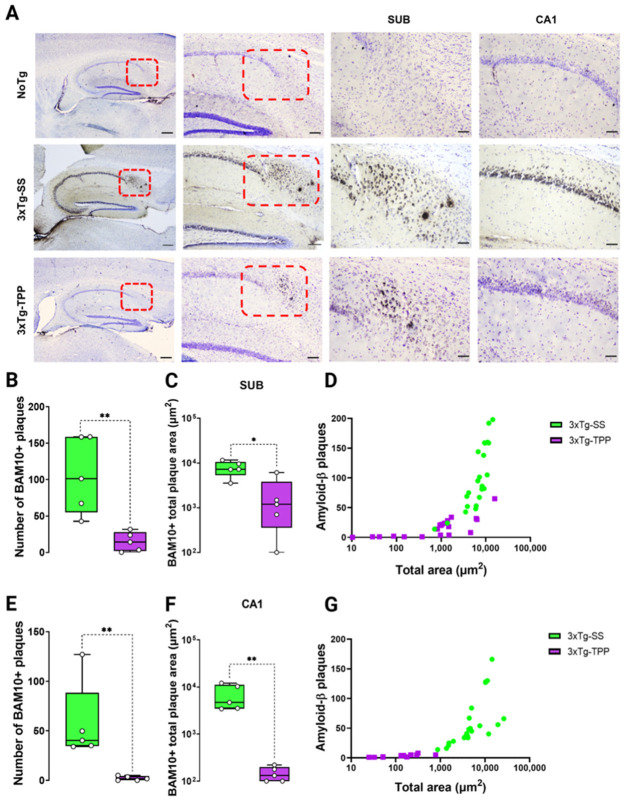
Hippocampal Aβ burden following TPP exposure in 3xTg-AD mice. (**A**) Representative images of the hippocampal formation and higher-magnification photomicrographs of the SUB and CA1 immunostained with the anti-Aβ antibody BAM10 in NoTg, 3xTg-SS (9 mg/mL), and 3xTg-TPP (2 mg/mL) mice. Scale bars: 500 μm (40×), 100 μm (100×), and 50 μm (200×). BAM10+ labeling was absent in NoTg animals and was observed in both intracellular and extracellular compartments in 3xTg-SS mice. Lower BAM10+ labeling was observed in the SUB and CA1 of 3xTg-TPP mice compared with 3xTg-SS mice. (**B**) Number of BAM10+ plaques in the SUB. (**C**) Total BAM10+ plaque area in the SUB. (**D**) Two-dimensional plot showing plaque number versus area in the SUB. (**E**) Number of BAM10+ plaques in the CA1. (**F**) Total BAM10+ plaque area in the CA1. (**G**) Two-dimensional plot showing plaque number versus area in the CA1. Data are presented as median and interquartile range. Asterisks indicate significant differences between groups after the Mann–Whitney U test (* *p* < 0.05, ** *p* < 0.01, *n* = 5 animals/group). Abbreviations: SUB, subiculum; Aβ, amyloid-β; NoTg, non-transgenic; SS, saline solution; TPP, thiamine pyrophosphate.

Amyloid burden was quantified to evaluate whether chronic TPP exposure was associated with changes in Aβ accumulation within early-affected hippocampal regions of 3xTg-AD mice. Quantification of BAM10+ immunolabeling showed fewer Aβ-positive plaques in the subiculum (SUB; U = 0, *p* = 0.012) and CA1 (U = 0, *p* = 0.0079) of 3xTg-TPP mice compared with 3xTg-SS mice. This pattern was consistent with the lower Aβ-immunostaining labeling area observed in both regions. As expected, BAM10+ labeling was detected in 3xTg-AD mice but not in NoTg animals (Figure 2A). Box and whisker plots summarize plaque number and total plaque area in SUB (U = 1, *p* = 0.015) and CA1 (U = 0, *p* = 0.008), as well as 2D analysis of plaque number versus area of Aβ plaques (Figure 2B–G; Supplementary Materials Figure S1). Data are presented as median and interquartile range. Statistical differences between 3xTg-SS and 3xTg-TPP groups were evaluated using the Mann–Whitney U test (** *p* < 0.01, *n* = 5 animals/group).

### 2.3. Microglial Density in the Hippocampal Formation of 3xTg-AD Mice Following TPP Exposure

**Figure 3:**
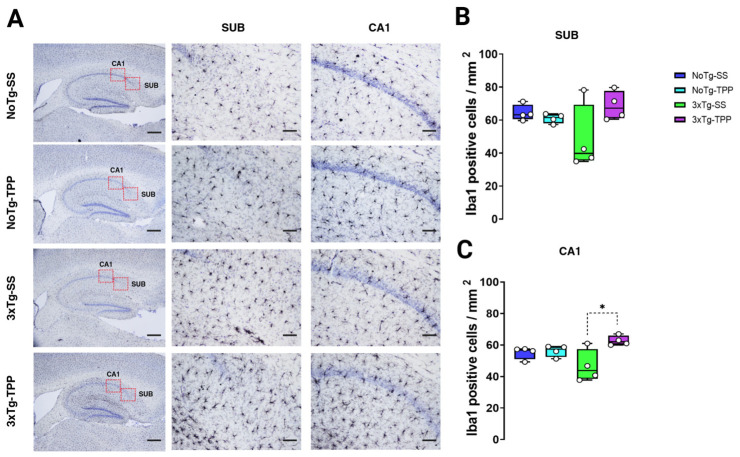
Microglial density in the hippocampal formation of 3xTg-AD mice following TPP exposure. (**A**) Representative images of the hippocampal formation and higher-magnification photomicrographs of SUB and CA1 from Iba1-immunostained brain slices of NoTg and 3xTg-AD mice receiving saline solution (SS, 9 mg/mL) or thiamine pyrophosphate (TPP, 2 mg/mL) for 42 days. Scale bars: 500 μm (40×) and 50 μm (200×). (**B**) A box-and-whisker plot showing the density of Iba1+ cells/mm^2^ in the SUB. (**C**) A box-and-whisker plot showing the density of Iba1+ cells/mm^2^ in the CA1. Microglial counts were normalized by area (mm^2^). Data are presented as median and interquartile range. Asterisks indicate significant pairwise differences identified by Dunn’s post hoc test following the Kruskal–Wallis test (* *p* < 0.05, *n* = 4 animals/group). Abbreviations: SUB, subiculum; CA1, Cornu Ammonis 1; Aβ, amyloid-β; NoTg, non-transgenic; SS, saline solution; TPP, thiamine pyrophosphate.

Microglial density was quantified to determine whether chronic TPP exposure was associated with changes in the abundance and regional distribution of microglial cells within the hippocampal formation of 3xTg-AD mice. Quantification of Iba1+ cells/mm^2^ showed that overall microglial density numbers remained similar across groups, with a difference detected only in CA1 (Figure 3A,C). Kruskal–Wallis test revealed a group effect in CA1 (H (3) = 8.356, *p* = 0.022), and Dunn’s post hoc test indicated a higher number of Iba1+ cells in 3xTg-TPP mice compared with 3xTg-SS (*p* = 0.036). In contrast, no significant differences were detected in the SUB (H (3) = 3.822, *p* = 0.297). Data are presented as median and interquartile range. Asterisks indicate significant pairwise differences identified by Dunn’s post hoc test following Kruskal–Wallis analysis (*p < 0.05, *n* = 4 animals/group).

These observations indicate that hippocampal microglial density remained relatively stable across groups at this stage of pathology, with a region-specific difference detected in CA1 following TPP.

### 2.4. Microglial Morphology and Structural Complexity in the Hippocampal Formation of 3xTg-AD Mice Following TPP Exposure

**Figure 4:**
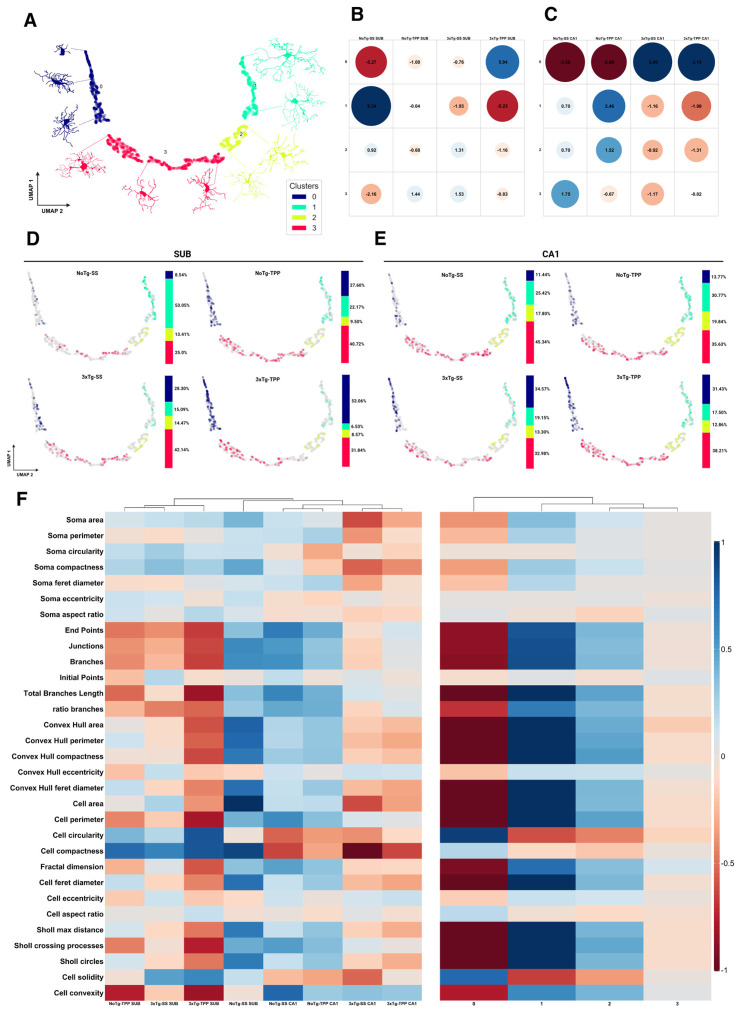
Microglial morphology and structural complexity in the hippocampal formation of 3xTg-AD mice following TPP exposure. (**A**) Representative microglial morphologies corresponding to the four clusters identified by the MorphoGlia pipeline. Cluster 0 comprised less complex morphologies, Cluster 1 included highly ramified cells, and Clusters 2 and 3 displayed intermediate profiles. (**B**,**C**) Standardized residuals from the chi-square analysis showing the distribution of microglial clusters in the SUB (**B**) and CA1 (**C**). Blue circles indicate positive residuals and red circles indicate negative residuals. Color intensity and circle size are proportional to the magnitude of the standardized residuals. (**D**,**E**) UMAP plots illustrating the relative distribution of microglial clusters across experimental groups in the SUB (**D**) and CA1 (**E**). (**F**) Heatmaps comparing experimental groups and clusters based on 32 extracted morphological features. The heatmap organized by experimental group (**left**) shows differences in branching, branch length, Sholl complexity, and other morphometric parameters according to genotype, treatment, and hippocampal region. The cluster-based heatmap (**right**) highlights the distinct morphometric profiles underlying each cluster. Abbreviations: SUB, subiculum; UMAP, Uniform Manifold Approximation and Projection; NoTg, non-transgenic; SS, saline solution.

Microglial morphology was analyzed to evaluate whether chronic TPP exposure was associated with differences in structural complexity in 3xTg-AD mice (Figure 4A–F; Supplementary Materials Figures S2–S4). Using the MorphoGlia pipeline developed by Maya-Arteaga and colleagues [35], 1740 microglial cells were analyzed and classified into four clusters (Clusters 0–3) spanning a continuum from less complex morphologies with reduced branching and lower Sholl complexity (Cluster 0) to highly complex and ramified cells with greater structural complexity (Cluster 1), whereas Clusters 2 and 3 displayed intermediate profiles (Figure 4F). Across hippocampal regions, 3xTg-AD mice were enriched in Cluster 0, whereas NoTg mice showed a greater representation of Cluster 1 cells. Following TPP exposure, differences in cluster distribution were detected across groups and regions (χ^2^ = 270.78, *p* < 2.68 × 10^−45^), while region-specific associations were identified by standardized residual analysis (Figure 4B,C).

In the SUB, NoTg-SS mice showed a predominance of Cluster 1 cells, whereas NoTg-TPP mice displayed a broader distribution across Clusters 1–3. In contrast, 3xTg-SS mice were enriched in Cluster 0. 3xTg-TPP mice exhibited a distribution characterized mainly by Clusters 0 and 3, indicating a shift toward less complex and intermediate morphologies in this region (Figure 4D). In the CA1, NoTg-SS mice showed a greater proportion of Cluster 3 cells, whereas NoTg-TPP mice were enriched in Clusters 1 and 2. 3xTg-SS mice remained predominantly associated with Cluster 0. In contrast, 3xTg-TPP mice exhibited an intermediate cluster distribution, with representation of Cluster 0 together with a greater contribution of Cluster 2 relative to 3xTg-SS mice. (Figure 4E).

Heatmap of morphometric parameters further distinguished the identified clusters and experimental groups (Figure 4F). In the SUB, differences were primarily observed in branching, number of initiation points, and Sholl complexity, with 3xT-SS animals showing lower values than NoTg groups. In CA1, NoTg-TPP mice were associated with greater branching, total branch length, and cell convexity, whereas 3xTg-SS mice retained less complex morphometric profiles. 3xTg-TPP mice showed intermediate morphometric profiles in CA1, overlapping features of Clusters 0 and 2. Together, these observations indicate that microglial morphology differed across hippocampal subregions and experimental conditions, with the transgenic background associated with reduced structural complexity.

### 2.5. Descriptive Analysis of Inflammatory and Metabolic Gene Expression in the Hippocampus Following Chronic TPP Exposure

**Figure 5:**
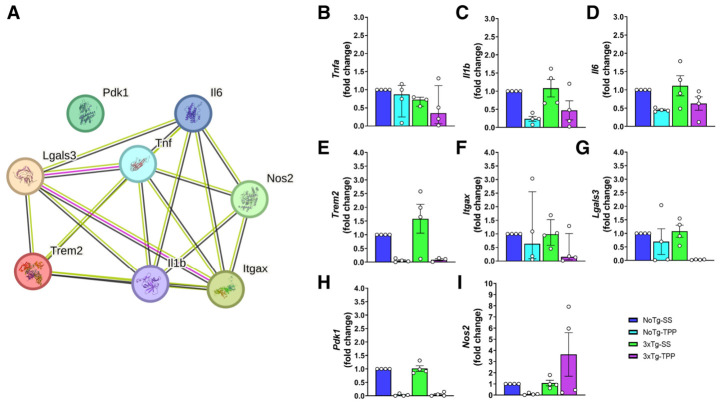
Descriptive, inflammatory, and metabolic relative gene expression in the hippocampus following TPP exposure in NoTg and 3xTg-AD mice. (**A**) Interactome network of microglia-related genes generated using STRING (https://string-db.org; accessed on 8 February 2026). Nodes represent proteins: colored nodes are query proteins and first-shell interactors. Edges represent protein–protein associations derived from different sources of evidence. Green edges indicate text-mining associations, pink edges indicate experimentally determined interactions, and black edges indicate co-expression relationships. Hippocampal mRNA expression levels were quantified by RT-qPCR and expressed as fold change calculated using the 2^−ΔΔCt^ method, normalized to the geometric mean of RPL13A and RPL27 as housekeeping genes. Data are presented as median and interquartile range (*n* = 3–4 animals per group). Due to the limited number of biological replicates, results are shown descriptively. (**B**) *Tnf,* (**C**) *Il1b*, (**D**) *Il6*, (**E**) *Trem2*, (**F**) *Itgax*, (**G**) *Lgals3*, (**H**) *Pdk1*, and (**I**) *Nos2* relative mRNA expression levels. Abbreviations: RT-qPCR, reverse transcription quantitative polymerase chain reaction; TNF, tumor necrosis factor.

To explore whether chronic TPP exposure was associated with differences in the expression of genes related to microglial activation and metabolism, hippocampal mRNA levels were analyzed by reverse transcription quantitative polymerase chain reaction (RT-qPCR) (Figure 5A). To examine potential functional relationships among the analyzed genes, an interactome network was generated using STRING (https://string-db.org; accessed on 8 February 2026). The resulting network showed interactions among inflammatory mediators (*Tnf*, *Il1b*, *Il6*, *Nos2*), microglial receptors (*Trem2*), and metabolic regulators (*Pdk1, Lgals3*), indicating that these genes participate in interconnected pathways relevant to microglial activation and neuroinflammation (Figure 5A).

Descriptive comparison of relative mRNA expression levels (2^−ΔΔCt^) revealed variability across experimental groups (Figure 5B–I). Data are presented as median and interquartile range to illustrate central tendency and dispersion within each group. In NoTg mice, the NoTg-TPP group showed lower median expression of Il1b, Il6, Trem2, Nos2, and Pdk1 compared with NoTg-SS controls. Lgals3 and Pdk1 displayed lower median values in 3xTg-TPP than in 3xTg-SS mice. In contrast, Nos2 expression showed higher median levels in 3xTg-SS mice than in other groups.

Overall, gene expression distributions in 3xTg-SS mice were broadly similar to those observed in NoTg controls for several markers, although variability differed among genes. Given the limited number of biological replicates (*n* = 3–4 per group), no inferential statistical analyses were performed, and results are presented descriptively. These findings are exploratory and hypothesis-generating.

## 3. Discussion

The present study provides a descriptive and region-specific characterization of hippocampal Aβ burden, microglial morphology, and selected metabolic gene expression following chronic TPP exposure in female 3xTg-AD mice. Rather than establishing direct causal mechanisms, these findings identify coordinated patterns consistent with links between thiamine-dependent metabolic pathways and microglial diversity, as described in experimental models of Alzheimer’s disease.

Alzheimer’s is recognized as a multicausal disease in which the development of effective therapies remains challenging. Key contributing processes include tau pathology, Aβ aggregation, neuroinflammation, synaptic dysfunction, and metabolic pathways [36]. From this perspective, preclinical and clinical evidence supports the therapeutic potential of thiamine derivatives in AD. Thus, benfotiamine, a lipophilic thiamine derivative, has been shown to improve spatial memory, reduce Aβ deposition and tau phosphorylation, and increase thiamine levels in cortical areas of transgenic AD mice [32,37]. Unlike benfotiamine, which requires phosphorylation by TPK1 to generate active TPP, direct administration of TPP bypasses this enzymatic step [23]. Because TPK1 expression is reduced in AD brains [27], direct TPP administration may theoretically bypass a potential metabolic bottleneck that could limit the efficacy of thiamine precursors.

In addition to its metabolic role, thiamine pyrophosphate may also be relevant in glial biology. Thiamine pyrophosphatase (TPPase) activity has been identified in microglial cells, localized to the plasma membrane and cellular processes, suggesting active thiamine-related metabolism in these cells [38]. This supports the notion that thiamine-dependent pathways may contribute to microglial function, providing a conceptual framework to interpret the patterns observed following TPP exposure in the present study.

Among the principal findings of this study were lower median *Pdk1* and *Lgals3* transcript levels in TPP-exposed groups, together with lower hippocampal Aβ burden. PDK1 phosphorylates and inhibits pyruvate dehydrogenase, thereby limiting pyruvate’s entry into the TCA cycle and favoring glycolytic metabolic programs previously associated with inflammatory microglial states [14]. Therefore, the lower median *Pdk1* expression observed following TPP exposure is consistent with transcriptional patterns previously associated with greater reliance on oxidative phosphorylation, although metabolic flux was not directly assessed. Similarly, lower median *Lgals3* expression may be relevant because galectin-3 has been associated with impaired Aβ clearance and neuroinflammatory responses in AD [15,39].

Consistent with these transcriptional patterns, BAM10+ immunoreactivity revealed lower Aβ burden in both SUB and CA1, accompanied by a shift toward smaller and more compact deposits. PCA-based analyses (Supplementary Figure S1) further suggested differences in plaque size and structural compactness [7]. Together, these observations are compatible with previous reports linking thiamine-dependent metabolic pathways with amyloid dynamics in vulnerable hippocampal circuits; however, they do not establish direct mechanistic modulation [27,30]. Although amyloid burden was quantitatively assessed using BAM10+ immunohistochemistry, future studies incorporating biochemical analyses of soluble and insoluble Aβ species, as well as APP-processing pathways, will be important for further characterizing the effects of TPP exposure.

On the other hand, morphometric clustering identified four microglial populations representing a continuum from less complex to highly ramified morphologies. Across regions, 3xTg-AD mice were predominantly associated with low-complexity clusters, whereas NoTg mice showed greater representation of highly ramified morphologies. Following TPP exposure, microglial distributions differed across hippocampal subregions. In the SUB, 3xTg-TPP mice showed greater representation of low-complexity and intermediate clusters, whereas in CA1, the distribution was more similar to that observed in 3xTg-SS mice.

In the central nervous system, thiamine has been implicated in regulating microglial immune responses and bioenergetic programs relevant to AD [40,41,42]. Thiamine deficiency has been associated with increased microglial proliferation and neuronal loss in rodent brains [31,43], whereas increased numbers of Iba1+ microglia have been reported in the hippocampus of 3xTg-AD mice from six months of age [44]. In the present study, overall, Iba1+ cell density was largely comparable between NoTg and 3xTg-AD mice; however, TPP-exposed 3xTg-AD animals displayed higher microglial numbers in the CA1. Given that thiamine has been associated with microglial bioenergetics and responsiveness [10,41,42], this regional increase may be compatible with altered microglial recruitment or persistence in CA1, although cell-specific functional assays were not performed.

TPP exposure was associated with region-specific shifts in microglial morphological clusters. In the SUB, 3xTg-TPP mice showed a higher relative abundance of low-complexity microglia together with a lower extracellular Aβ burden. Low-complexity morphologies have previously been associated with reactive, migratory, and phagocytic states in neurodegenerative contexts [45,46,47]. By contrast, NoTg animals were enriched for highly ramified clusters, which are commonly associated with surveillance-related phenotypes [35,45,47]. Intermediate clusters may represent transitional morphologies along the continuum between reactive and homeostatic states.

In contrast, microglial cluster distributions in CA1 were more similar between TPP and SS-treated 3xTg-AD mice, although 3xTg-TPP animals showed a modest increase in intermediate complexity clusters relative to 3xTg-SS mice. This regional difference may reflect distinct influences of extracellular versus intracellular Aβ accumulation on microglial structural plasticity, since extracellular plaque pathology is more prominent in the SUB, whereas intracellular Aβ is still predominant in CA1 at this age in 3xTg-AD mice [44,48,49]. TPP exposure was also associated with subtle morphological shifts in NoTg mice, although the functional significance of these changes remains uncertain [50]. Overall, these findings suggest that TPP exposure may be associated with changes in the relative representation of distinct microglial states rather than with a uniform morphological response. However, morphology alone does not allow direct functional inference.

Our histological observations were aligned with the gene expression distributions. Relative to NoTg-SS mice, 3xTg-SS animals showed differences in the expression of inflammatory and metabolism-related genes, which may reflect distinct microglial states within the AD model. In contrast, TPP exposure was associated with lower median expression of Pdk1 and Lgals3, as well as lower hippocampal Aβ burden. These findings are consistent with previous studies implicating thiamine-dependent pathways in microglial metabolism and amyloid-associated inflammation [14,27,41].

PDK1 phosphorylates and inactivates PDH, thereby restricting pyruvate entry into the TCA cycle and promoting glycolytic metabolism [14,27]. Because glycolytic programs have been associated with pro-inflammatory microglial states, the lower median Pdk1 expression observed in TPP-treated groups may be compatible with transcriptional patterns previously associated with greater reliance on OXPHOS and metabolic function [41,51,52]. Similarly, lower median Lgals3 expression may be relevant, as galectin-3 is a TREM2 ligand associated with impaired Aβ clearance and inflammatory microglial activation in AD [15,39]. TPP-exposed groups also showed lower median expression of IL-1β, IL-6, Trem2, and Nos2 relative to saline controls. Together, these transcriptional patterns may be compatible with previously described immunometabolic frameworks in which thiamine availability influences the balance between inflammatory and homeostatic microglial programs [10,14,17].

These observations should nevertheless be interpreted cautiously. RT-qPCR analyses were performed with a limited number of biological replicates; therefore, the transcriptional findings are descriptive rather than inferential. In addition, neither metabolic flux nor cell-type-specific gene expression was directly assessed. Consequently, the present data do not demonstrate that TPP directly modifies microglial metabolism or function, but rather identify transcriptional patterns that are compatible with mechanisms proposed in previous studies [14,19,41].

In addition, the present study identified morphological, amyloid-associated, and transcriptional changes following TPP exposure. Additional protein-level characterization using markers such as CD68, TREM2, TMEM119, P2RY12, or Galectin-3, together with amyloid-related co-localization approaches, will be necessary to further define the functional phenotypes and microglial states associated with TPP exposure [17,39].

Compared with previous studies using high-dose oral benfotiamine, six weeks of continuous low-dose TPP administration (2 mg/mL) was associated with detectable histological and transcriptional differences. Because TPP is the active form of thiamine, direct administration may bypass metabolic limitations associated with reduced TPK1 expression observed in AD brains [23,27,53]. In line with prior observations of thiamine-related enzymatic activity, such as TPPase, in microglial cells, this may also be compatible with cell-type–specific metabolic engagement [38]. However, the present study did not directly compare TPP with benfotiamine; therefore, no conclusion regarding relative efficacy can be drawn.

Overall, these findings suggest that thiamine-dependent metabolic pathways may contribute to microglial and amyloid-related changes in the hippocampus of 3xTg-AD mice. Future studies combining dose–response approaches, metabolic flux analyses, and cell-specific profiling will be necessary to determine whether these descriptive associations reflect direct mechanisms relevant to AD progression.

## 4. Materials and Methods

### 4.1. Experimental Animals

Homozygous 9-month-old female 3xTg-AD mice (harboring APPSwe and tauP301L transgenes on a mutant PS1M146V knock-in background) generated from a B6129S hybrid background (*n* = 20) were used, as well as NoTg mice of the same age and sex. Animals were maintained under the same B6129S hybrid background (*n* = 20). All mice were bred and raised in the vivarium laboratory of the Institute of Neurobiology (INB)-UNAM, housed in polycarbonate cages (12 × 12 × 25 cm) under standard conditions (4–5 animals per cage, 20–25 °C, 40–70% humidity, 12 h light/dark cycle) with ad libitum access to Purina^®^ Chow 5001 and water. 3xTg-AD and NoTg mice (129C57/BL6) were randomly assigned to experimental groups (TPP treatment or SS, saline solution) using a simple randomization procedure prior to the start of the intervention. All subsequent experimental procedures and analyses were conducted using the assigned group codes.

Only female mice were used for this study, since sex-dependent differences in the 3xTg-AD model are well documented. Females show an earlier onset of amyloid and tau pathology compared to males [48,54]. Regarding age, nine months is considered “early middle-age” in mice. According to research, this is a suitable window to study early pathological changes and therapeutic interventions [55]. During early middle age, 3xTg-AD females exhibit evident intracellular Aβ accumulation in the CA1 and SUB. Extracellular plaques emerge later, around 11–12 months of age [56]. Importantly, cognitive alterations and diffuse Aβ deposits have also been reported at nine months of age [57], supporting its selection as an appropriate time point for intervention. The estrous cycle was monitored, and females were preferentially tested and processed during the diestrus phase to reduce hormonal variability [58].

All procedures were approved by the Bioethics Committee of the Faculty of Medicine, Universidad Autónoma de Querétaro (FM-UAQ, protocol number 12853, date of approval: 28 October 2021) and complied with the institutional guidelines of the INB-UNAM (protocol number 057, date of approval: 28 February 2013) and international standards for the care and use of laboratory animals (NIH). To minimize experimental bias, all procedures—from surgical implantation to behavioral testing and immunohistochemical analysis—were conducted under blinded conditions. Clinical trial number: not applicable.

### 4.2. Pharmacological Intervention

A stock solution of TPP was prepared at a final concentration of 2 mg/mL in sterile saline [59]. Continuous delivery was achieved using Osmotic pumps model 2006 (ALZET^®^, Cupertino, CA, USA) (0.15 μL/h for 42 days), corresponding to an estimated cumulative exposure of approximately 12.1 mg/kg in mice weighing ~25 g. The required amount of TPP was dissolved in 0.9% saline, gently mixed until homogeneous, and immediately loaded into the osmotic pumps according to the manufacturer’s instructions. For vehicle treatments, only saline was administered. Four experimental groups were used: transgenic mice treated with TPP (3xTg-TPP, *n* = 10), transgenic mice treated with vehicle solution (3xTg-SS, *n* = 10), and non-transgenic mice treated with TPP and SS as controls (NoTg-TPP, *n* = 10; NoTg-SS, *n* = 10). A three-centimeter surgical incision was made along the middorsal plane. Afterward, the skin was separated from the muscle, and the pump was inserted beneath the skin. After wound closure, a topical analgesic-antibiotic ointment was applied to prevent self-scratching and infection [60]. Mice were allowed to recover from anesthesia for 30–60 min on a heating pad and then returned to their cages under continuous monitoring for the first 48 h, the critical period for pump activation. The implanted pump contained either TPP or vehicle solution and was weighed before filling and after completion of the procedure to verify solution delivery. For surgery, the animals were anesthetized using a 70:30 mixture of ketamine and xylazine at a dose of 70 mg/kg and 6 mg/kg, respectively. TPP (X-2) was obtained from the Instituto de Investigaciones Filosóficas y Científicas, S.A. de C.V. Drug delivery was performed using osmotic pumps (model 2006, ALZET^®^, Cupertino, CA, USA), which provide continuous release via an osmotic mechanism. In this process, water influx through a semipermeable membrane generates pressure that drives the drug solution at a constant rate (0.15 μL/h, 42 days).

### 4.3. Nest Shredding and Nest Building Test

The nest-building task (*n* = 10 animals per group) was assessed during the final week of treatment, after the animals had undergone habituation and handling. This assay evaluates innate, hippocampal-dependent behavior in rodents and is widely used as a sensitive indicator of cognitive function and general well-being [33,34]. For the test, each mouse was individually housed in a clean polycarbonate cage (12 cm × 12 cm × 25 cm) with food and water available ad libitum. A single pressed cotton pad (5cm × 5 cm) was placed in the lower left corner of the cage. This nestlet was provided at the beginning of the dark phase of the reversed light/dark cycle, allowing the animals to manipulate it for 24 h. After this period, the nests were photographed from above, and any unused material was removed. Nest quality was scored according to a 1–5 scale (1 = no nest; 5 = satisfactory nest), as described by Deacon [34]. Scoring was performed independently by two blinded observers to minimize bias. Both the final score and the material used were included in the statistical analysis.

### 4.4. Tissue Preparation

Brain tissue was collected at the humanitarian endpoint and processed through two distinct procedures. For histological and immunohistochemical analyses, animals were deeply anesthetized with an overdose of sodium pentobarbital (0.22 mL/kg) administered intraperitoneally. Adequate anesthesia depth was confirmed by the absence of response to tail pinch stimulation prior to intracardiac perfusion. Mice were transcardially perfused with phosphate-buffered saline (PBS; 30–50 mL, room temperature) for 1–2 min, followed by 4% paraformaldehyde (PFA). Brains were post-fixed in 4% PFA for 24 h, cryoprotected in 30% sucrose in PBS for 48 h, and sagittal-sectioned using a Leica CM1510 cryostat (Leica Biosystems, Nussloch, Germany) for subsequent staining and imaging.

For molecular analyses, animals were euthanized by rapid decapitation without perfusion; the hippocampus was immediately dissected from fresh tissue, snap-frozen on dry ice, and preserved with RNAlater™ Solution (AM7020, Thermo Fisher Scientific, Waltham, MA, USA) for RNA stabilization and storage at −80 °C. All procedures were approved by the Bioethics Committee of the INB-UNAM (protocol code 057) and conducted in accordance with the Mexican Official Standard for the Use and Care of Laboratory Animals (NOM-062-ZOO-1999) and the National Research Council guidelines (NRC, 2010).

### 4.5. Immunohistochemistry

After 24 h in post-fixation in 4% PFA, the tissue was moved to a 30% (m/v) sucrose solution for 48 h for cryoprotection. Subsequently, a Leica CM1510 cryostat (Leica Biosystems, Nussloch, Germany) was used to freeze the tissue and cut it at a thickness of 40 µm. Four to five sections from the left or right hemisphere of each animal were selected according to the Paxinos and Franklin mouse brain atlas [61]. Hippocampal sections corresponding approximately to −1.34 to −2.30 mm relative to bregma were analyzed. Standardized anatomical regions corresponding to the CA1 and SUB hippocampal areas were evaluated. Images were acquired at 200× magnification using identical acquisition parameters, and the full image field was used for subsequent analysis. For each animal, one image per region was acquired from 4 to 5 sections, and values obtained from the corresponding image fields were averaged to generate a single biological value per animal prior to statistical analysis, thereby avoiding pseudoreplication. Sections were then washed under free-floating conditions in 0.1 M PBS for 10 min and incubated in citrate buffer (pH = 6.0) at 80–100 °C for 20 min to expose epitopes, followed by three 10 min washes in 0.1 M PBS (pH = 7.4) containing 0.05% Tween 20. Afterward, sections were incubated overnight with 0.1 M Iba1 antibody (1:500; ab107159, RRID: AB_10972670; Abcam, Cambridge, UK) while shaking at 4 °C. The next day, immunolabeled brain sections were incubated with a biotinylated anti-goat secondary antibody (1:500; BA-9500, RRID: AB_2336123; Vector Laboratories, Newark, CA, USA) for 2 h, followed by a signal amplification step. Visualization was achieved using the diaminobenzidine-glucose oxidase method, as previously described [35]. For the amyloid antibody, we used 90% formic acid for 5 min, then washed quickly in distilled water, followed by three 10 min washes in 0.1 M PBS (pH 7.4) containing 0.05% Tween 20. Afterward, sections were incubated overnight with 0.1 M BAM10 (1:500; A3981, RRID: AB_1078153; Sigma-Aldrich, St. Louis, MO, USA) while shaking at 4 °C. The next day, immunolabeled brain sections were incubated with biotinylated anti-mouse secondary antibody (1:500; BA-2001-ZC1230, RRID: AB_2336180; Vector Laboratories, Newark, CA, USA)for 2 h. This was followed by an amplification signal step. For visualization, the diaminobenzidine-glucose oxidase method was used, as previously described. Additionally, we performed three 10 min washes in 0.1 M PBS (pH = 7.4) containing 0.05% Tween 20. The sections were mounted on slides in 0.05 M PBS, dehydrated in xylene, and subsequently covered with Entellan^®^ (Merck, KGaA; Darmstadt, Germany).

### 4.6. Morphological Analysis

Photomicrographs of BAM10+ plaques and Iba1+ cells in the SUB and CA1 of the hippocampal formation were acquired using a Nikon Eclipse Ci-Li light microscope (Nikon Corporation, Tokyo, Japan) equipped with a Nikon Plan 40x/0.65 air lens (*n* = 4 animals per group) and the NIS-Elements Viewer software (Version 4.13.00, Nikon Corporation, Tokyo, Japan). Digital images were pre-processed under blinded conditions using Fiji/ImageJ software (version 1.53c; National Institutes of Health, Bethesda, MD, USA; https://imagej.net/ij (accessed on 8 February 2026)), based on previous protocols [35,62]. Resulting binary images in TIFF format were analyzed using MorphoGlia, a computational tool for automated analysis and unbiased classification of microglial morphology.

The MorphoGlia pipeline is an unsupervised framework grounded in manifold learning. The pipeline employs recursive feature elimination to identify the most informative morphometric descriptors and reduce redundancy in the feature space. The selected features are subsequently embedded using Uniform Manifold Approximation and Projection (UMAP), which represents the data as a fuzzy topological graph while preserving both local and global relationships on a low-dimensional manifold. Finally, clustering is performed on 1740 microglial cells using Hierarchical Density-Based Spatial Clustering of Applications with Noise (HDBSCAN), a density-based approach capable of detecting clusters of variable shapes and sizes without requiring a predefined number of clusters [35].

For the UMAP parameters, nearest neighbors (n_neighbors) were set to 20, and the minimum distance between points (min_dist) = 0.05. For the HDBSCAN, minimum cluster size (min_cluster_size) = 30 and minimum cluster samples (min_samples) = 30. Color-coded cells were generated and mapped back onto the tissue microphotograph to visualize their spatial arrangement. 

For the BAM10+ plaque analysis, digital micrographs were acquired under identical microscope settings and processed using Fiji/ImageJ software (version 1.53c; National Institutes of Health, Bethesda, MD, USA; https://imagej.net/ij (accessed on 8 February 2026)) under blinded conditions. Images were converted to grayscale and subjected to color deconvolution to isolate the signal. A uniform Otsu threshold was set to lower = 0 and upper = 190, and applied across groups to define BAM10+ immunoreactivity. Plaques were quantified using the Analyze Particles function, excluding background artifacts by setting a minimum size (µm^2^): >0–10,000 µm^2^ and a circularity range of 0.20–1.00.

### 4.7. RNA Extraction and Quantitative Reverse Transcription Polymerase Chain Reaction (RT-qPCR)

Total RNA was extracted from brain tissue homogenized by maceration. To do so, the TRIzol^®^ Reagent method (15596018, Invitrogen, Carlsbad, CA, USA) was followed according to the manufacturer’s instructions. TRIzol^®^ Reagent (1 mL) was added for every 100 mg of sample, then the sample was incubated for 5 min at room temperature. Subsequently, 0.2 mL of chloroform was added for each milliliter of TRIzol^®^ Reagent used. The aqueous phase was centrifuged and recovered in a new tube. Next, 0.5 mL of isopropanol was added for each milliliter of TRIzol^®^ Reagent used, incubated for 10 min at room temperature, and then centrifuged. RNA purity and yield were determined by measuring the absorbance ratio at 260/280 nm in a NanoDrop™ ND-2000 spectrophotometer (Thermo Fisher Scientific, Waltham, MA, USA). To perform total RNA electrophoresis, a 1.5% agarose gel was made and stained with SYBR green. The obtained RNA was used to carry out retrotranscription and cDNA was synthesized using the RevertAid^®^ First Strand cDNA Synthesis Kit (K1621, Thermo Fisher Scientific, Waltham, MA, USA) according to the supplier’s instructions. The cDNA was stored at –70 °C until analysis by polymerase chain reaction (PCR).

Amplification was performed using a StepOne^®^ thermal cycler (Thermo Fisher Scientific, Waltham, MA, USA), and data were collected using StepOne Software v2.3™ (Thermo Fisher Scientific, Waltham, MA, USA). All primer sequences utilized to identify the fragments of the genes of interest were designed with a similar Tm (Table 1). Therefore, RT-qPCR conditions were as follows: final reaction volume of 10 ul; alignment temperature of 62 °C; a holding stage of 95 °C for 10 min; a cycling stage consisting of 40 cycles at 95 °C for 15 s, 62 °C for 40 s, and 72 °C for 10 s; a melting curve stage of 95 °C for 15 s, 60 °C for 1 min, and 95 °C for 15 s. Rpl27 (F: 5′-TGGCTGGAATTGACCGCTAT-3′; R: 5′-TTCAAAGCTGGGTCCCTGAA-3′) and Rpl13A (F: 5′-GTGGAAGTACCAGGCAGTGA-3′; R: 5′-AGGAGTCCGTTGGTCTTGAG-3′) were used as controls for normalization.

### 4.8. Statistical Analysis

For behavioral analysis, there were 10 animals per group; for histological analyses, four to five animals per group; and for RT-qPCR analyses, three to four animals per group. Each animal served as a unit of analysis. For histology, two fields (SUB, CA1) and four to five sections per animal were quantified and averaged to produce a single value per animal. For RT-qPCR, three technical replicates were averaged per animal. Statistical analyses and graphical representations were performed using GraphPad Prism 8 (GraphPad Software, La Jolla, CA, USA) and R version 4.4.0 (R Foundation for Statistical Computing, Vienna, Austria). Data distribution was first assessed with the D’Agostino–Pearson omnibus test. Non-parametric data were expressed as median and interquartile range. Statistical significance was indicated only when differences were observed relative to the control group: * *p* < 0.05; ** *p* < 0.01. Nest-building behavior was analyzed using a Kruskal–Wallis test followed by Dunn’s post hoc test for multiple group comparisons. For histological analyses, which included quantification of BAM10+ plaques and Iba1+ cells per mm^2^ in the SUB and CA1 regions, we used the Mann–Whitney U test for comparisons between two groups and the Kruskal–Wallis test followed by Dunn’s post hoc test for multiple-group comparisons. Microglial morphology was assessed using MorphoGlia [35], which integrates dimensionality reduction with UMAP and supervised clustering with HDBSCAN. Cluster frequencies across experimental groups and hippocampal subregions were analyzed with Pearson’s chi-square test, and standardized residuals and heatmaps were generated to visualize associations between clusters and experimental conditions. Finally, hippocampal gene expression profiles obtained from RT-qPCR were calculated using the 2^−ΔΔCt^ method with the geometric mean of RPL13A and RPL27 as housekeeping genes [68]. Statistical comparisons were performed using the Kruskal–Wallis test followed by Dunn’s *post hoc* test.

## 5. Limitations

Our study has several limitations. The small number of biological replicates per group (*n* = 3–4) limits statistical power and generalizability; therefore, findings should be interpreted with caution. Metabolic flux, enzymatic activity, and microglial functional responses were not directly assessed, and interpretations rely on transcriptional and morphological associations. Only female 3xTg-AD mice were included, and a single TPP dose and treatment duration were evaluated. Future studies incorporating larger cohorts, dose–response analyses, and functional approaches will be required to further define the role of thiamine-dependent pathways in neurodegenerative contexts.

## 6. Conclusions

In summary, this study describes region-specific hippocampal patterns involving microglial heterogeneity, amyloid burden, and thiamine-associated transcriptional profiles in 3xTg-AD female mice. While descriptive, these findings provide a framework for future investigations addressing metabolic regulation within AD-related neuroinflammatory contexts.

## Figures and Tables

**Table 1:** Microglial activation and metabolism-related genes evaluated in the hippocampus of wild-type and 3xTg-AD mice treated with TPP or saline solution.

Gene	Codified Protein	Functions	Forward (F)/Reverse(R) Sequence
*Trem2*	TREM2	Involved in microglial activation, proliferation, migration, apoptosis, and expression of pro-inflammatory cytokines [10].	F: 5′-AAGTGGAACACAGCACCTCC-3′ R: 5′-AGGATCTGAAGTTGGTGCCC-3′
*Lgals3*	GAL3	Highly expressed in AD, mainly in the brain when damaged by reactive microglia; interacts with immune receptors like TREM2 [39].	F: 5′-TCCACTTTAACCCCCGCTTC-3′ R: 5′-AGTTGGCTGATTTCCCGGAG-3′
*Itgax*	Cd11c	Counteracts Aβ deposition via increased Aβ-uptake and degradation by microglia [63].	F: 5′-TCGGGTAGCATCAGTTCCAC-3′ R: 5′-TGTGTACCCTCTTAGCTGCC-3′
*Il1b*	IL-1β	Mediates microglial activation and proliferation [64].	F: 5′-GTAATGAAAGACGGCACACCC-3′ R: 5′-TCCTGACCACTGTTGTTTCCC-3′
*Il6*	IL-6	Induces microglial activation, cell proliferation, and the production of microglia-derived pro-inflammatory molecules [65].	F: 5′-AGACAAAGCCAGAGTCCTTCAG-3′ R: 5′-GAGCATTGGAAATTGGGGTAGG-3′.
*Tnf*	TNFα	Involved in chronic neuroinflammation mediated by microglia; regulates morphological changes and cytokine release, and controls the expression of neuroprotective factors [66].	F: 5′-AGTTCTATGGCCCAGACCCT-3′ R: 5′-ACAAGGTACAACCCATCGGC-3′.
*Nos2*	iNOS	It is Ca^2+^-independent and can be upregulated in response to TNF and IFNγ, or downregulated in response to IL-1β; involved in nitric oxide production [67].	F: 5′-CCCAGCCTTGCATCCTCATT-3′ R: 5′-TCTCTTGCGGACCATCTCCT-3′.
*Pdk1*	PDK1	Involved in facilitating the expression of inflammatory mediators and improving glucose uptake to support the glycolytic shift in reactive microglial phenotypes [19].	F: 5′-GGATTGCCCATATCACGCCT-3′ R: 5′-TCATGTCTTTCGGCTCTCGG-3′.

## Data Availability

All data generated or analyzed during this study are included in this published article. Supplemental Information can be found online. Further information and requests for resources and reagents should be directed to and will be fulfilled by the lead contact, S.D.-C. (yoldi@unam.mx) or P.G.-S. (pablo.garcia@uaq.mx).

## Supplementary Materials

The following supporting information can be downloaded at: https://www.mdpi.com/article/10.3390/ijms27115022/s1.
