# Supplementary material for: Hippocampal, Microglial, Morphological, and Amyloid Profiles Following Thiamine Pyrophosphate Treatment in 3xTg-AD Mice
Source: Int J Mol Sci. 2026 Jun 2;27(11):5022. doi: 10.3390/ijms27115022 (PMC13257033; doi:10.3390/ijms27115022)

SUPPLEMENTARY INFORMATION

Hippocampal Microglial Morphological and Amyloid Profiles Following Thiamine Pyrophosphate Treatment in 3xTg-AD Mice

Nelly Jovana Pastén-Castrejón[1,2], Humberto Martínez-Orozco[2], Gloria Yareli Gutiérrez-Silerio[1], Hebert Luis Hernández-Montiel[3], Juan Pablo Maya-Arteaga[2], Israel Poblano-Paez[2], Pablo García-Solís[1*], Sofía Yolanda Díaz-Miranda[2*].

1 Laboratorio de Endocrinología y Nutrición, Facultad de Medicina, Centro de Investigación Biomédica Avanzada de la Universidad Autónoma de Querétaro, Santiago de Querétaro 76140, México; jovana.pasten.08@gmail.com (N.J.P.-C.) gloriagutierrezsilerio93@gmail.com (G.Y.G.-S.) pablo.garcia@uaq.mx (P.G.-S.)

2 Laboratorio de Neuromorfometría y Desarrollo, Departamento de Neurobiología del Desarrollo y Neurofisiología. Instituto de Neurobiología, Universidad Nacional Autónoma de México, Querétaro, 76230, México; israelpoblano9015@outlook.com (I.P.-P.); juanpablo.maya@hotmail.com (J.P.M.-A.) h_martinez@live.com.mx (H.M.-O.); yoldi@unam.mx (S.Y.D.-M.);

3 Laboratorio de Neurobiología y Bioingeniería Celular, Facultad de Ciencias Naturales de la Universidad Autónoma de Querétaro, Querétaro, 76230, México; hebert@uaq.mx (H.L.H.-M.)

* Correspondence: pablo.garcia@uaq.mx Tel.: +52 (442)-192-12-00 Ext.: 62530 (P.G.S.); yoldi@unam.mx Tel.: +52 (55)-56-23-40-58 Ext.: 34058 (S.Y.D.-M.)

SUPPLEMENTARY INFORMATION

Table of Contents

page

Figure S1. PCA-based characterization of BAM10^{+} plaque morphology in SUB and CA1. This figure summarizes the PCA-based analysis of BAM10^{+} plaque morphology in the SUB and CA1 regions, highlighting group-dependent differences in plaque size and compactness, and visualizing the contribution of each morphological feature through combined PCA loadings. 3

Figure S2. Image pre-processing of brain tissue. A) Image pre-processing of immunostained microglia Iba-1+ in brain tissue. B) Binary image of Iba-1 + cells processed using Image J Fiji. C-D) Cluster analysis and grouping of cells according to their morphological features with MorphoGlia. 4

Figure S3. Dynamic selection of the most appropriate features to characterize microglia in the subiculum and CA1 subregions of 3xTg-AD mice using Recursive Feature Elimination (RFE) algorithm in MorphoGlia. 5

Figure S4. Cluster cell count shows the absolute number of cells in each cluster for the study groups in SUB (A) and CA1 (B). The quantity and proportion of cells change acord to the experimental group after the microglial characterization with MorphoGlia. 6

Figure S1. PCA-based characterization of BAM10⁺ plaque morphology in SUB and CA1. PCA of BAM10+ plaque morphological parameters in (A) SUB and (B) CA1. Each point represents an individual plaque: 3xTg-SS (green circles) and 3xTg-TPP (purple squares). PC1 was primarily associated with plaque size, while PC2 reflected differences in shape (circularity and solidity). In both regions, TPP treatment shifted the distribution toward smaller, morphologically more compact plaques, with a more pronounced separation in CA1. (C) Heatmap of the combined PCA loadings (SUB+CA1). PC1 was dominated by size parameters (Area, Major, Minor), while PC2 reflected differences in shape (Circularity, Solidity). The combined pattern indicates that the overall morphological variation of the BAM10+ plates is structured primarily along axes of size and compactness, which were modulated by the TPP treatment.

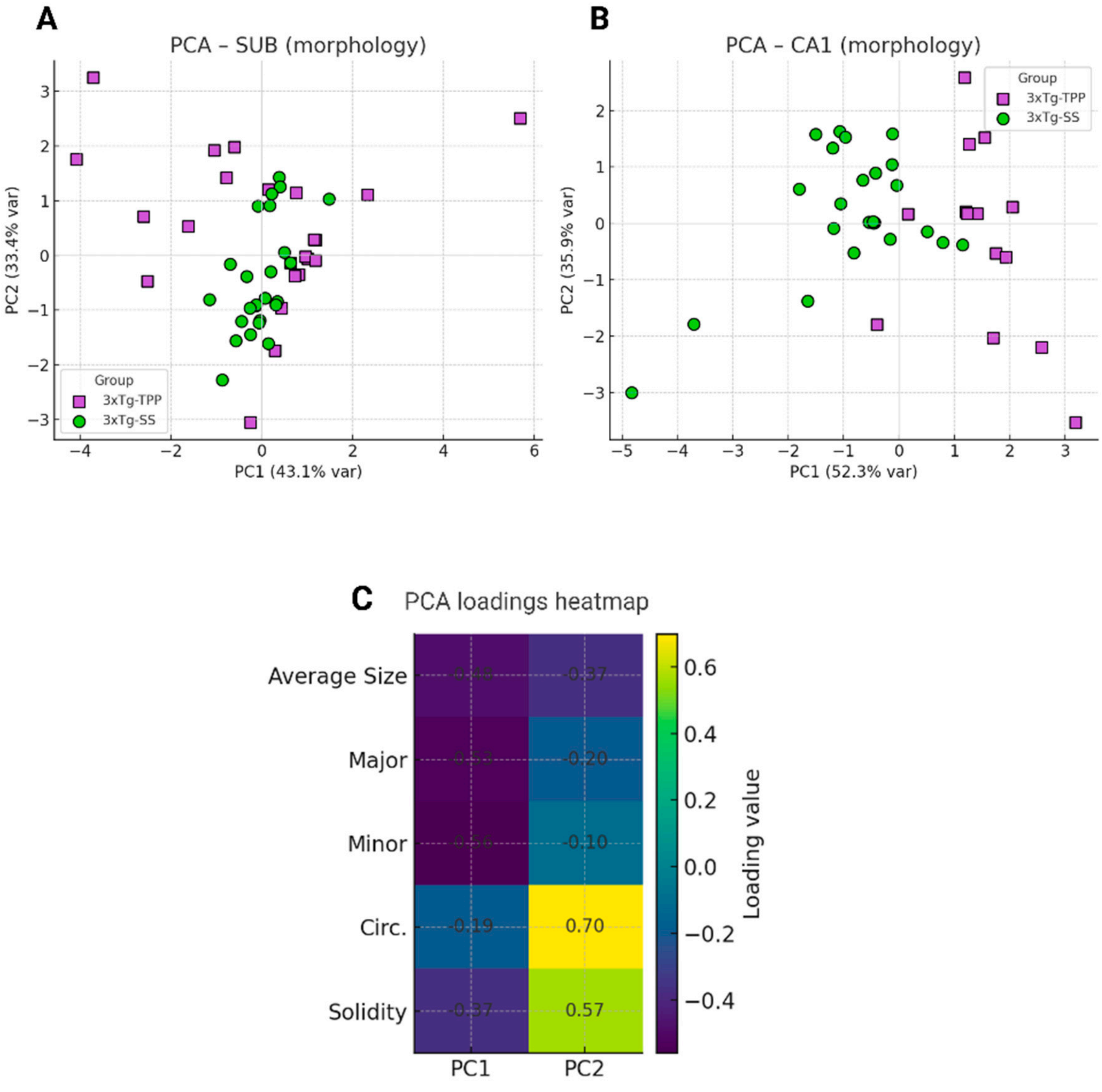

Figure S2. Image pre-processing of brain tissue. A) Image pre-processing of immunostained microglia Iba-1+ in the brain tissue. B) Binary image of Iba-1 + cells processed using Image J Fiji. C-D) Cluster analysis and grouping of cells according to their morphological features with Morphoglia.
Representative image-processing workflow used for BAM10+ plaque analysis in hippocampal sections. Images were acquired at 200× magnification, and the full field corresponding to the SUB region or the immediately adjacent CA1 area was analyzed. Scale bars were intentionally omitted in this workflow illustration to avoid interference with thresholding and segmentation procedures.

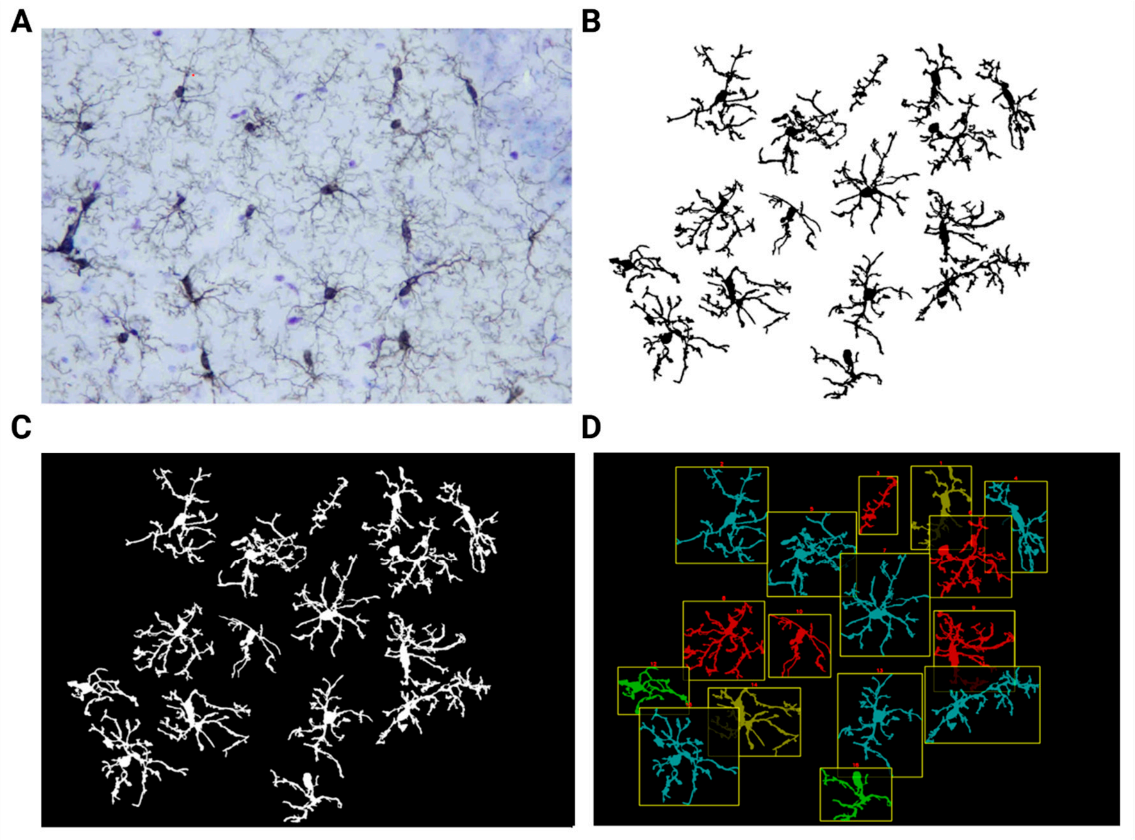

Figure S3. Dynamic selection of the most appropriate features to characterize microglia in the subiculum and CA1 subregions of 3xTg-AD mice using Recursive Feature Elimination (RFE) algorithm in MorphoGlia.

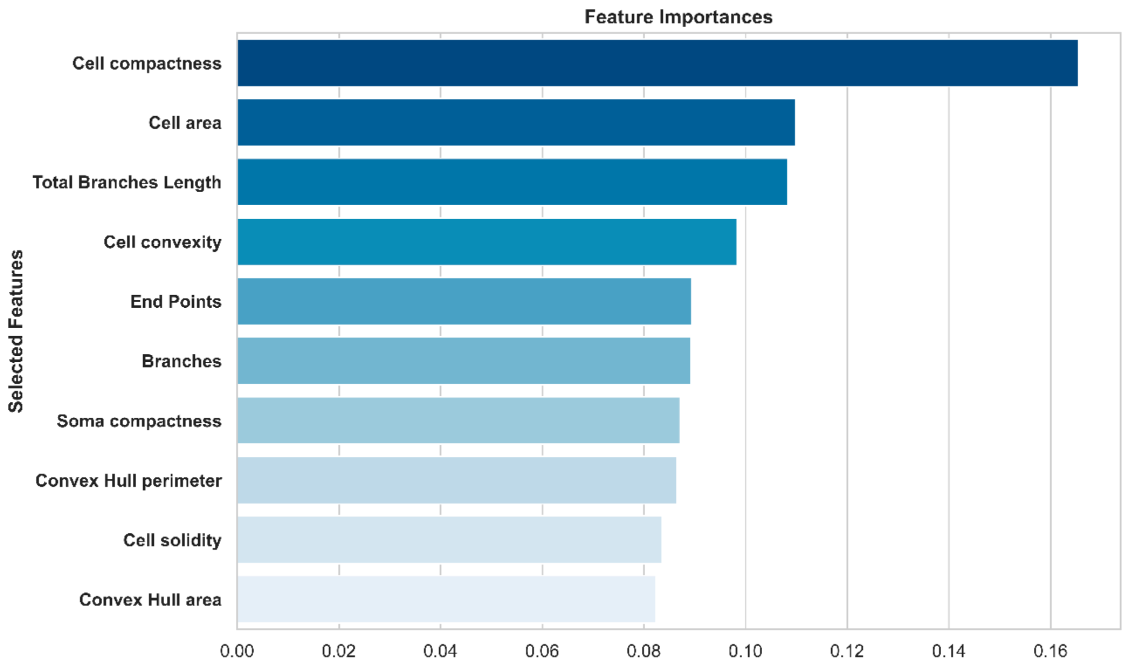

Figure S4. Cluster cell count shows the absolute number of cells in each cluster for the study groups in SUB (A) and CA1 (B). The quantity and proportion of cells change acord to the experimental group after the microglial characterization with MorphoGlia.

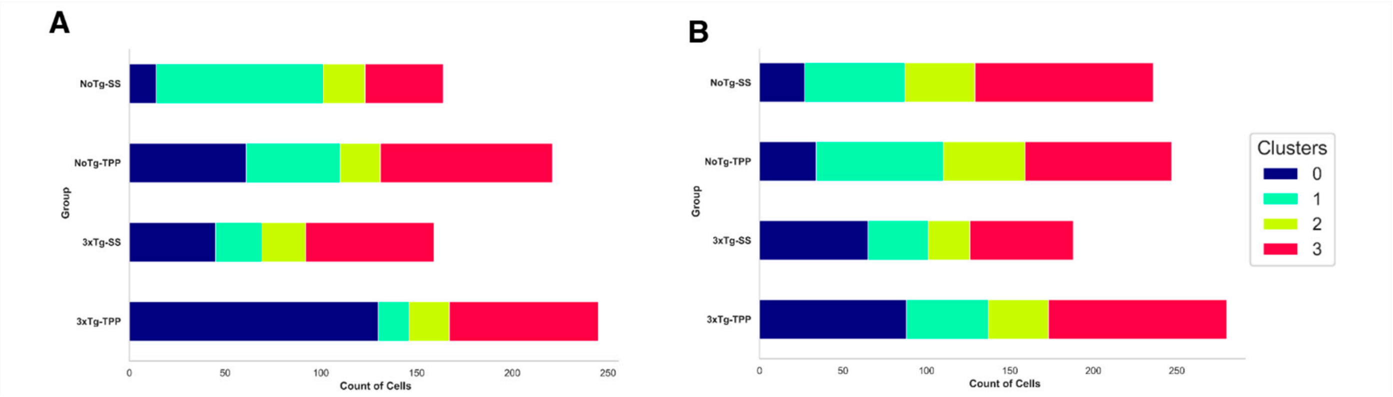

Supplement: Supplementary file 1 [file ijms-27-05022-s001.zip › ijms-4320477-supplementary.pdf]
